# Updating beliefs beyond the here-and-now: the counter-factual self in anosognosia for hemiplegia

**DOI:** 10.1093/braincomms/fcab098

**Published:** 2021-05-21

**Authors:** Louise P Kirsch, Christoph Mathys, Christina Papadaki, Penelope Talelli, Karl Friston, Valentina Moro, Aikaterini Fotopoulou

**Affiliations:** 1 Institut des Systèmes Intelligents et de Robotique, Sorbonne Université, Paris 75005, France; 2 Department of Clinical, Educational and Health Psychology, University College London, London WC1E 6BT, UK; 3 Scuola Internazionale Superiore di Studi Avanzati (SISSA), Trieste 34136, Italy; 4 Interacting Minds Centre, Aarhus University, Aarhus 8000, Denmark; 5 Translational Neuromodeling Unit (TNU), Institute for Biomedical Engineering, University of Zurich and ETH Zurich, Zurich 8032, Switzerland; 6 Homerton Hospital, London E9 6SR, UK; 7 Wellcome Centre for Human Neuroimaging, Institute of Neurology, University College London, London WC1N 3AR, UK; 8 NPSY.Lab-VR, Department of Human Sciences, University of Verona, Verona 37129, Italy

**Keywords:** belief-updating, motor awareness, anosognosia, right-hemisphere stroke, metacognition

## Abstract

The syndrome of anosognosia for hemiplegia, or the lack of awareness for one’s paralysis following right hemisphere stroke, can provide unique insights into the neurocognitive mechanisms of self-awareness. Yet it remains unclear whether anosognosia for hemiplegia is a modality-specific deficit of sensorimotor monitoring, or whether domain-general processes of attention and belief-updating converge to cause anosognosia for hemiplegia. Using a Bayesian learning framework, we formalized and empirically investigated the hypothesis that failures to update anosognosic beliefs can be explained by abnormalities in the relative uncertainty (i.e. precision) ascribed to prior beliefs versus sensory information in different contexts. We designed a new motor belief-updating task that manipulated both the temporal (prospective and retrospective) and spatial (hemispace most affected by inattention and hemispace less affected by inattention) conditions in which beliefs had to be updated, and we validated its sensitivity to anosognosia for hemiplegia in 26 patients with right hemisphere stroke. We then computed and empirically tested two different Bayesian predictors of prospective beliefs using two proxies for precision in anosognosia for hemiplegia patients: (i) standardized, neuropsychological measures of objective attention abilities, i.e. visuospatial neglect scores and (ii) subjective uncertainty reports, i.e. confidence ratings. Our results suggest that while neglect does not affect local, sensorimotor error monitoring, it does seem to affect the degree to which observed errors are used to update more general, prospective beliefs about counterfactual motor abilities in anosognosia for hemiplegia. Difficulties in such ‘counterfactual’ belief-updating were associated with disruptions in tracts of the ventral attentional network (i.e. superior longitudinal fasciculus connecting the temporo-parietal junction and ventral frontal cortex) and associated lesions to the insula, inferior parietal cortex and superior temporal regions. These results suggest that self-awareness extends beyond local, retrospective monitoring, requiring also salience-based, convergence of beliefs about the self that go beyond the ‘here-and-now’ of sensorimotor experience.

## Teaser

Kirsch et al. developed a novel task to manipulate the temporal (prospective and retrospective) and spatial context of motor belief-updating under a Bayesian modelling framework, finding that salience-related, right hemisphere disconnections affect individual’s ability to evaluate the relevance of context-dependent sensorimotor errors to more abstract (context-independent) beliefs about the self.

## Graphical Abstract

**Graphical Abstract:**
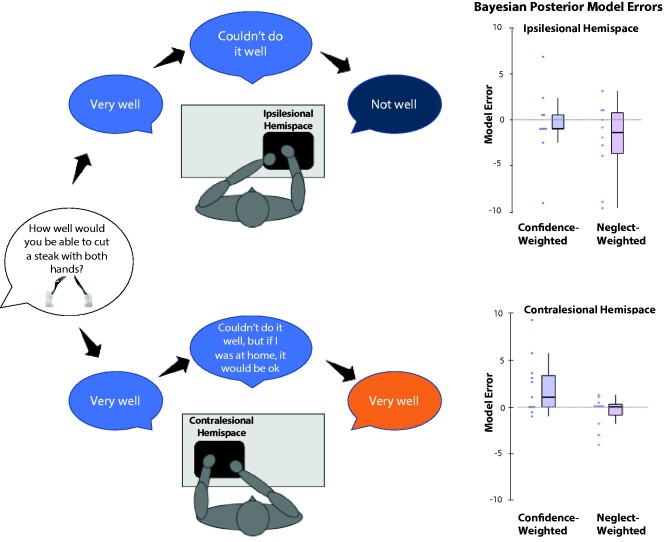

## Introduction

In order to navigate a changing world, people have to update their current beliefs in the face of new evidence. This belief-updating may be facilitated by the monitoring and evaluation of experience, as when one notices one’s mistakes without any feedback. This ability is typically referred to as metacognition.^1^^,^^2^ People may have different degrees of confidence (subjective uncertainty) about the accuracy of their perceptions or memories—and a large body of scientific evidence is dedicated to such ‘retrospective’ aspects of metacognition.^3^ People may also vary in how they metacognitively evaluate their abilities in the future. For example, people may be overconfident in their ability to learn new skills. This ‘prospective’ metacognition has been shown to dissociate from retrospective metacognition.^3^ Finally, people may also have different evidence gathering strategies regarding their beliefs, prioritizing certain pieces of evidence over others; for example, paying more attention to confirmatory positive or negative information.^4^^,^^5^ Such differences in belief-updating and metacognition have been of great interest across many fields, spanning from social sciences to neuroscience and psychiatry, with several debates such as the relation between the above kinds of metacognition, the Bayesian-optimality of such processes^6^ and their domain-general or modality-specific nature.^7^ Although metacognitive beliefs are an important part of the construct of metacognition, we can note that the terminology encompasses other processes as well (e.g. there are ongoing debates about the precise relation between metamemory and mentalisation to perceptual metacognition).

A unique way to inform such debates is to systematically study deficits in belief-updating in neurological patients. One such neuropsychological symptom is anosognosia for hemiplegia (AHP), defined as the apparent unawareness of one’s paralysis,^8^ which occurs typically following stroke-induced right perisylvian lesions.^9^ It is axiomatic to AHP that patients fail to update their beliefs regarding their motor abilities even when confronted with their severe, contralesional motor loss during neurological examination.^10^^,^^11^ Moreover, patients do not update their anosognosic beliefs even against the evidence of their massively compromised daily living abilities, more frequent falls than other stroke patients^12^ and ample medical and social feedback.^13^ In that sense, their beliefs are considered as delusional. Despite advances in the understanding of AHP,^14^ at least two fundamental questions remain regarding the inability of these patients to update their delusional beliefs about themselves.

First, it is debated whether AHP can be explained as a secondary consequence of concomitant sensory or cognitive deficits, or whether it is a specific disorder of forward, motor monitoring. According to the former, patients with AHP are unable to update their beliefs regarding their motor abilities because they no longer have good enough access to contrary ‘feedback’ about their paralysis. For example, patients may be unable to notice their errors and update their beliefs due to their contralesional neglect.^15^ By contrast, according to action monitoring theories,^16^^,^^17^ patients with AHP have a specific deficit in monitoring the discrepancy between predicted and actual sensory feedback due to lesions to the lateral premotor cortex.^18^^,^^19^ In support of this theory, we have experimentally observed that patients experience an illusory sense of moving their paralyzed arm only when planning to move themselves and not when they are anticipating passive movement of the same arm, planned by someone else.^20^

Second, it is unclear whether patients’ inability to update their anosognosic beliefs is modality-specific (local monitoring deficits), or whether domain-general processes of belief-updating and metacognition are also necessary to account for AHP (global monitoring deficits).^21–24^ Patients with AHP have reality monitoring deficits, confusing for example merely imagined with actually executed actions^25^^,^^26^ and belief-updating deficits, being for instance overconfident and inflexible in a verbal information-gathering.^27^ Finally, anosognosic errors are associated with disruptions of allocentric mentalisation related to inferior parietal lobule lesions,^13^ suggesting that patients’ self-awareness is not facilitated by the ability to ‘see’ themselves as others regard them, similarly to findings in other neuropathologies and psychopathologies.^28–31^

Taken together, accumulated evidence suggests that anosognosic beliefs for hemiplegia (HP) can be explained by both impaired local sensorimotor monitoring and impairments in more global, metacognitive monitoring. Such multicomponent understandings of anosognosic behaviours in general can be found also in other fields such as in dementia research.^28^ However, previous multifactorial models of AHP have considered the relation between such factors as merely cumulative, with damage to at least two independent modules considered necessary for AHP to occur.^21^^,^^23^^,^^32^ Using a unifying theoretical framework (the Bayesian Brain hypothesis),^33^^,^^34^ we have proposed instead that AHP can be explained as a disconnection between several of the normally convergent sensorimotor, metacognitive and mentalisation functions that support self-awareness,^23^^,^^24^^,^^35^ as explained below.

According to this framework, the brain uses its prior learning to construct generative models about the embodied self that encode predictions not only about the hidden causes of current, noisy sensory inputs, but also about the inferred causes of ‘counterfactual’ sensory inputs. The latter depend on predicted but not-as-yet executed actions (e.g. what will it feel like when I grab that cup of hot coffee), potential spatial positions one may occupy (e.g. how would I grab that cup of coffee if I were sitting at the other side of the table), emotional and social conditions one may encounter (e.g. how embarrassed would I be if my friend saw me drop that coffee cup).^24^^,^^36^ In that sense, self-awareness involves inferential processes with counterfactual depth.^24^^,^^37^ Accordingly, the inability of patients to update their anosognosic beliefs may be understood as the inability to draw new inferences not only about their motor abilities in the here-and-now of experience (e.g. did I just move as I intended to?), but also about counterfactual, prospective motor abilities (e.g. could I do this same action tomorrow, or at home?). To our knowledge, however, this kind of prospective awareness has not been yet examined in AHP.

Furthermore, according to this Bayesian brain hypothesis framework, belief-updating is dependent upon the relative *uncertainty* (or, mathematically its inverse *precision*)^38^^,^^39^ ascribed to prior beliefs relative to sensory information, which determines how prediction errors are weighted in the formation of posterior beliefs. In computational psychiatry, precision abnormalities have provided an explanation for psychopathological symptoms, including delusions.^40–42^ Accordingly, using a Bayesian learning framework,^43^^,^^44^ we formalized and empirically investigated the hypothesis that failures to update anosognosic beliefs about counterfactual motor abilities will be explained by abnormalities in the precision ascribed to prior beliefs relative to sensory information.^24^ Owing to the clinical restrictions of studying acute stroke patients, one cannot design a belief-updating task with sufficient trials to allow formal learning modelling as in many other psychopathologies. However, one can sample explicit beliefs under carefully controlled experimental conditions and use key neuropsychological measures as ‘proxies for precision’ to test alternative Bayesian models of belief-updating.

To this aim, we designed a new motor belief-updating task that manipulated both the temporal (prospective and retrospective) and spatial (affected versus unaffected hemispace) conditions in which beliefs had to be updated. The task allowed us to measure how prospective estimates about bimanual motor abilities are updated on the basis of retrospective estimates about corresponding action attempts in the contralesional (most affected by neglect) and in the ipsilesional (less affected by neglect) hemispace. The task also allowed us to compute and empirically test two different Bayesian predictors of prospective beliefs using two proxies for precision: (i) Objective standardized, neuropsychological measures of attention, i.e. visuospatial neglect scores and (ii) Subjective uncertainty reports, i.e. confidence ratings. We explain below the background and precise hypotheses that motivated these measures and manipulations.

Although patients with AHP typically also suffer from hemispatial neglect, neglect is not considered a necessary, nor sufficient deficit for AHP, given the long-observed double-dissociations between the two symptoms.^45^ However, such dissociations do not exclude the possibility that visuospatial neglect contributes to AHP in functional convergence with other deficits.^22^^,^^23^ In the framework used here, this functional convergence can be understood as related to precision. Specifically, in predictive coding, the precision afforded by various beliefs—or sensory evidence—can be taken as the computational homologue of attention.^46^^,^^47^ For example, attending to a particular source of information corresponds to increasing the precision of the associated (sensory) prediction errors. Thus, a formal account of visuospatial neglect—in terms of aberrant precision may—be particularly apt for explaining its contribution to anosognosia, as it has been in a related phenomenology of altered motor awareness, namely functional motor disorders.^48^ In such pathologies, precision optimization is regarded as a domain-general ability depending broadly on the functional convergence of various neuromodulatory functions.^49^ Yet in the case of AHP, the observed lesions and structural disconnections of the ventral attentional system,^13^^,^^50^ which have been linked with difficulties in reorienting attention in contralesional hemispace based on salience and behavioural relevance,^51^^,^^52^ may play a similar role, particularly when there are concomitant lesions to the basal ganglia and the limbic system.^50^^,^^53^^,^^54^

Accordingly, using a spatial manipulation and standardized measurements of each patient’s attentional deficits (as proxies for precision), we could generate an approximate measure of each patient’s ability to attend to prediction errors in the affected, contralesional versus the unaffected, ipsilesional hemispace. We hypothesized that AHP patients would have greater difficulties in monitoring their errors retrospectively and updating their beliefs prospectively in the affected than the unaffected hemispace, where their inattention would render prediction errors imprecise and would thus influence the relative precision of prior beliefs and sensory prediction errors.

Moreover, as neglect is not a sufficient explanation for AHP, we reasoned that measurements of subjective uncertainty^3^ could offer additional insights regarding the confidence with which patients hold their prior versus their retrospective, posterior beliefs. In at least some patients, other deficits could introduce biases in subjective uncertainty about beliefs,^27^ which in turn could cause performance monitoring errors as observed in other pathologies.^55^ Thus, we used confidence ratings as a second ‘subjective’ proxy for precision and tested whether neglect-based or, confidence-based Bayesian belief-updating models, including *objective* (neglect scores) versus *subjective* (confidence scores) proxies for precision, respectively, would best capture anosognosic beliefs in the different temporal and spatial conditions tested here.

Finally, we conducted voxel-based, lesion-symptom mapping analyses to identify the lesions and white matter disconnections associated with (i) clinical anosognosia; (ii) prior beliefs about motor ability across hemispaces; and (iii) posterior prospective beliefs in the contralesional hemispace, where we expected belief-updating difficulties. Consistently with the above disconnection hypotheses^23^^,^^24^ and based on previous work,^53^^,^^56^^,^^57^ including a recent study^50^ with an advanced lesion analyses methods and the largest sample to date (*N* = 174), we predicted that difficulties in updating prospective beliefs, particularly in the contralesional hemispace, will be associated with disruptions in tracts and structures belonging to at least two systems: namely the limbic system and the ventral attentional network [i.e. superior longitudinal fasciculus (SLF) connections between temporo-parietal junction and ventral frontal cortex, including in this case, possible direct lesions to the insula].

## Materials and methods

### Participants

Twenty-six, unilateral, right-hemisphere-lesioned stroke patients (mean age: 64.58 ± 14.26 years; 14 females) were recruited from consecutive admissions to seven stroke wards in London as part of a large study on body awareness after right hemisphere stroke using the following inclusion criteria: (i) imaging-confirmed first ever right hemisphere lesion; (ii) contralateral HP; (iii) <4 months from onset; (iv) no previous history of neurological or psychiatric illness; (v) >7 years of education; (vi) no medication with significant cognitive or mood side-effects; (vii) no language impairments that precluded completion of the study assessments; and (viii) right handed.

Patients were divided into two groups based on the presence (AHP group, *N* = 11) or absence (HP group, *N* = 15) of anosognosia for HP, diagnosed as in previous studies^13^^,^^56^ based on the Berti interview^10^ and validated using the Feinberg scale^58^ (see Supplementary materials, Methods 1, for full details).

All participants gave written, informed consent to participate in the study. The local National Health System Ethics Committees approved the study, which was carried out in accordance with the Declaration of Helsinki.

### Neuropsychological and neurological assessment

All patients underwent neurological and neuropsychological assessment, presented in Table 1. The two groups did not show significant differences in neuropsychological testing, other than in awareness measures, as expected. The target group showed a trend towards performing worse on the line bisection test. Beyond group differences, individual differences in neglect will be considered in subsequent analyses given our main spatial manipulation (see section below Bayesian Posterior Beliefs).

**Table 1:** Patient groups’ demographic and neuropsychological profiles

	AHP (*n* = 11)		HP (*n* = 15)		Mann–Whitney test		
	Mean	*SD*	Mean	*SD*	*Z*	df	*P*
Age (years)	66.82	13.600	61.87	15.226	−0.911	26	0.376
MRC Left upper limb (max 5)	0.09	0.302	0.08	0.289	−0.063	23	1.000
Berti motor awareness scale	2.364	0.9511	0.267	0.7761	−3.731	26	<0.001
Feinberg awareness scale	5.727	3.2432	1.208^c^	1.3392	−3.710	23	<0.001
Digit span forwards	6.45	1.368	6.23^b^	1.589	−0.446	24	0.665
Digit span backwards	3.73	1.421	3.92^b^	2.326	−0.474	24	0.656
MOCA memory (max 5)	1.75^e^	2.062	2.50^e^	1.773	−0.529	12	0.673
MOCA total (max 30)	17.35^e^	3.70	21.25^e^	5.59	−1.615	13	0.116
Personal bias (Comb/Razor bias)	−20.43^a^	27.89	−26.92^b^	36.74	−0.807	23	0.445
Line cancellation bias	49.21	49.18	−34.85^f^	43.21	−0.169	19	0.885
Bisiach one item test (max 3)	.38^c^	0.518	.22^e^	0.667	−1.039	17	0.506
Line bisection (max 9)	3.56^a^	2.242	5.92^b^	3.068	−1.990	22	0.048
HADS depression	7.00^d^	2.449	4.80^g^	3.271	−0.990	12	0.364
HADS anxiety	6.57^d^	1.988	7.20^g^	2.864	−0.423	12	0.711

Values calculated with missing data: a = group n-1; b = group n-2; c = group n-3; d= group n-4; e = group n-6; f = group n-7; g = group n-10. The Medical Research Council scale (MRC; Guarantors of Brain, 1986) was used to assess motor strength. The Berti motor awareness scale^10^ and the Feinberg awareness^58^ were used to assess anosognosia for hemiplegia symptoms. General cognitive functioning and long-term verbal recall were assessed using the Montreal Cognitive Assessment (MoCA)^59^ and working memory assessed using the digit span task from the Wechsler Adult Intelligence Scale III.^60^ Visuospatial neglect were assessed using subscales of the Behavioural Inattention Test^61^ (line cancellation and line bisection) and personal neglect was assessed by the ‘One Item’ test,^62^ and ‘Comb/Razor’ test.^63^ The Hospital Depression and Anxiety Scale (HADS)^64^ was used to assess anxiety and depression. The scores of both patient groups were within the normal range on the HADS (range: 0–7 normal, 8–10 borderline, 11+).

### Motor belief-updating task

#### Design and main predictions

**Figure 1:**
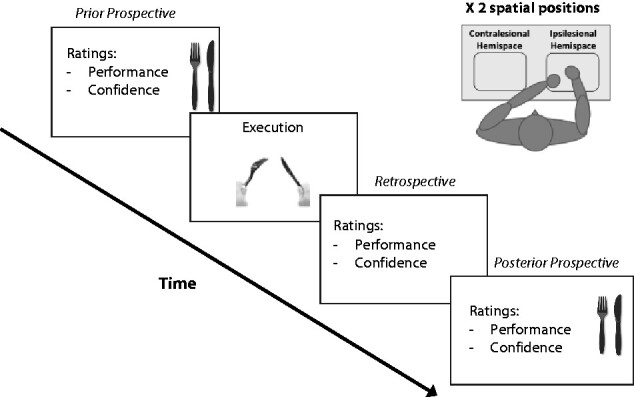
**Motor belief-updating task.** Timeline of one typical trial. Each patient performed the task once in each hemispace (contralesional and ipsilesional). Patients estimated their performance and confidence at three different time points: before attempting to execute the action (prior prospective estimates); just after the execution attempt (retrospective estimates); and a third time as posterior prospective estimates. Two different Bayesian update scores were computed from the patient’s ratings (see details in the Methods 2.4).

To quantify how anosognosic patients update their beliefs about motor abilities, we developed a new ‘Motor Belief-updating’ task (see Fig. 1). Participants were asked to estimate prospectively their motor ability to perform everyday bimanual actions, before attempts to execute such actions. After attempting the actions, patients had to then provide retrospective estimates of performance. Finally, they were asked again to estimate the corresponding motor ability prospectively. Thus, there were three estimates for each action, a prior prospective estimate, a retrospective estimate and a posterior prospective estimate. For each estimate, patients also provided a confidence rating, stating how confident they were in the accuracy of their estimate. Importantly, to manipulate the level of attention available to action monitoring, the requisite objects were presented in two different peripersonal spatial positions; namely, in the patient’s contralesional and ipsilesional hemispace, and patients had to attempt to perform the actions within these two hemispaces. This allowed us to first examine whether the two groups (patients with AHP and patients with HP) differed in their prior prospective beliefs about their ability to execute bimanual actions across the hemispaces (i.e. irrespective of neglect). Given their anosognosia, we expected the AHP group to show significantly higher scores than the HP group in both hemispaces, even though both groups were unable to perform any of the actions (due to their HP). This result would validate our task as a sensitive task for anosognosia. Correlations of prior and posterior prospective estimates with clinical AHP scores in both groups would add further validity to our task as capturing symptom-specific, belief-updating in patients with AHP.

We expected the AHP group to show greater difference between prior prospective and retrospective estimates, as well as between the latter and posterior prospective estimates, in the ipsilesional than in the contralesional field, suggesting that neglect influenced the degree they attended to their motor errors in the affected hemispace retrospectively and prospectively. Given the consecutive nature of the task, and individual variability in both neglect and subjective beliefs, to investigate how patients with AHP take into account the relative precision between their prior beliefs and their retrospective estimates when aiming to update their prospective beliefs, we formalized posterior prospective belief updating according to a Bayesian learning framework (see section Bayesian Posterior Beliefs below). This formalization allowed us to built alternative models of Bayesian prospective beliefs taking into account in each hemispace, each patient’s prior prospective belief (prior prospective estimates), evidence (retrospective estimates), and uncertainty (with two precision proxies: confidence as a subjective proxy vs. neglect as an objective proxy). We then compared these models against patients’ actual posterior estimates, under the hypothesis that the neglect-based model would capture posterior prospective beliefs better in the contralesional hemispace, whereas the confidence-based model would best explain ipsilesional hemispace beliefs.

#### Procedure and measures

To control for perceptual set and affordance, an object corresponding to a bimanual everyday action (e.g. using cutlery) was positioned on a table in front of the participants, on their midline, and they were asked to rate the importance of this action in their everyday life (e.g. ‘How important is it for you to be able to cut your steak using both hands?’ – note that no statistically significant differences were found between the groups, see Supplementary Results 1 for details). During the main task, participants were then presented with one of the objects (i.e. cutlery, or gloves, or shirt), positioned on the table in front of their torso, on pre-established distances (30 cm, either to the right or, to the left of the midline), corresponding to contralesional and ipsilesional hemispaces, as shown in Fig. 1.

Participants were then asked to give their motor performance estimates using an 11-point Likert-type ‘Performance scale’, vertically presented to avoid any confound with neglect, ranging from 0 = ‘Not at all’ to 10 = ‘Extremely well’. For every estimate, they were also asked to rate their confidence in the accuracy of their estimate using a six-point Likert-type, vertical ‘Confidence scale’ (0 = ‘Not at all confident’ to 5 = ‘Extremely confident’ – results for confidence ratings are presented in Supplementary Results 2 and Supplementary Fig. 1).

For each hemispace, one object was selected and patients were first asked for baseline Prior Prospective performance estimates and corresponding confidence ratings, as above (e.g. ‘Please give me your estimate about how well, in your current state, you would be able to put these gloves on both hands’).

The patient was subsequently asked to perform the action (e.g. put on a pair of gloves, hold a fork and knife, and pretend to cut a steak, button-up a shirt with both hands). They had a maximum of one minute to perform the action and could stop at any time. They were subsequently asked to rate their performance (Retrospective performance estimate, e.g. ‘How well have you done it?’), and to estimate their confidence, as above. Lastly, subsequently without any break, the experimenter asked participants to give a further, Posterior Prospective performance estimate, and a corresponding confidence rating (see Fig. 1; e.g. ‘Now that you’ve tried this, can you please give me your estimate again about how well, in your current state, you would be able to put these gloves on both hands’). Following a break of minimum 20–30 min, to avoid carry-over effect and allow some rest, the procedure was then repeated with a new item in the other hemispace (out of three, put on gloves, cut food with cutlery and button a shirt with both hands, randomized between participants and hemispaces, as much as possible due to the odd numbers). The order of the two blocks was counterbalanced between the participants in each group.

### Bayesian Posterior Beliefs

Posterior Prospective Performance estimates can be influenced by different types of *uncertainty* or *precision* which can influence the way patients weight the new incoming information (i.e. their perception of failure to perform the bimanual action, given by their retrospective performance estimate). Two Bayesian Posterior Beliefs were computed for each patient with AHP on each hemispace, with two indexes of uncertainty, in turn (i) patients’ confidence ratings on their performance estimates as a *subjective* proxy for precision and (ii) patients’ neglect scores as an *objective* proxy for precision:where $\mu_{\theta }$ = prior prospective motor performance estimate; *y* = retrospective motor performance estimate; $\pi_{\varepsilon }$ = retrospective confidence estimate, and $\pi_{\theta |y}$ = $\pi_{\varepsilon }+\pi_{\theta }$, with $\pi_{\theta }$= prior prospective confidence estimate.where $\mu_{\theta }$ = prior prospective motor performance estimate; y = retrospective motor performance estimate; and ν (neglect score) is the rescaled composite neglect score. This neglect score was computed by first rescaling separately the scores of the line cancellation and line bisection tests (subparts of the Behavioural Inattention Test)^61^ in order to have 0 = no bias and 100 = maximal bias, and then by averaging these two scores for each patient.

- **The confidence-weighted Bayesian posterior belief about motor ability** was computed as a measure considering both prior prospective and retrospective performance estimates as well as confidence ratings to calculate an updated Bayesian prospective motor belief. This measure was generated using the generic Bayesian update equation for beliefs in response to new information under a Gaussian model with conjugate prior.^43^^,^^44^ This measure allows us to assess how confidence-weighted, prior prospective motor beliefs are updated based on retrospective beliefs about one’s action attempt. Specifically, the confidence-weighted Bayesian posterior belief $\mu_{\theta |y\pi }$ was computed as follows:
  $$\;\mu_{\theta |y\pi }=\;\mu_{\theta }+\;\frac{\pi_{\varepsilon }}{\pi_{\theta |y}}(y-\mu_{\theta })$$

- **The neglect-weighted Bayesian posterior belief about motor ability** was computed as a measure considering both prior prospective and retrospective performance estimates as well as individual external precision, as measured by patients’ neglect scores. Specifically, the neglect-weighted Bayesian posterior belief $\mu_{\theta |yn}$was computed as follows:
  $$\mu_{\theta |yn}=\;\mu_{\theta }+\;\frac{1}{1+\nu (\mathrm{neglect}\mathrm{score})}(y-\mu_{\theta })$$

Finally, comparing these Bayesian posterior beliefs $\mu_{\theta |y\pi }$and $\mu_{\theta |yn}$ to the actual posterior prospective performance estimate given by AHP patients allows the measurement of whether patients estimated their performance in a Bayesian way given their prior prospective belief (prior prospective estimates), evidence (retrospective estimates) and uncertainty (precision proxies: confidence vs. neglect). These Bayesian posterior belief model errors (confidence-weighted and neglect-weighted, respectively) were independently computed in each hemispace, by subtracting each Bayesian posterior belief to the actual posterior prospective performance estimate patients gave.

### Behavioural statistical analyses

First, we assessed whether our task could capture the unawareness of motor disabilities in the AHP group. To do so, we compared prior prospective performance estimates in both AHP and HP groups. As these estimates were not normally distributed in the HP group, the effect of group was analysed using a non-parametric Mann–Whitney test; hemispace effects were analysed using a Wilcoxon Signed Rank Test; and the interaction of group and hemispace was analysed by calculating the difference between the estimates on the ipsilesional and the contralesional hemispace, using non-parametric Mann–Whitney tests to ask whether the group had a significant effect on this difference.

Given that the HP control groups’ prior belief scores were very low across hemispaces (ceiling effects) and hence they had little meaningful margins for belief-updating, we focussed our analyses on potential hemispace differences in the AHP group. For completeness, we conducted the corresponding, analyses with both groups and observed similar patterns of results (Supplementary Fig. 2 and Supplementary Results Section 3).

To examine hemispace differences in retrospective estimates, we compared prior prospective performance estimates to retrospective performance estimates in the AHP group, depending on the hemispace the action was performed. A 2 × 2 repeated-measures ANOVA was conducted as the residuals were approximately normally distributed. Similarly, we assessed prospective estimates, by comparing retrospective performance estimates to posterior perspective performance estimates, in the AHP group, depending on hemispace. Bonferroni corrections were used to correct for multiple comparisons in post-hoc tests. All reported values are two-tailed.

Finally, to investigate prospective belief updating following action, Bayesian posterior belief model errors were analysed in the AHP group, separately for each hemispace with one-sample *t*-tests to assess for any significant deviation of the posterior prospective performance estimate from the computed Bayesian posterior belief (different from 0), either taking into account the confidence in the performance estimates or patients’ neglect scores (see section Bayesian Posterior Beliefs above).

All behavioural analyses were conducted in SPSS23 (IBM Corp.) and JASP (JASP Team, 2020). Figures for behavioural data were generated in R (R Core Team, 2013), using ggplot2.^65^

### Lesion analyses

Voxel-based lesion symptom mapping (VLSM)^66^ was used for our main lesion analyses using as predictors (i) Prior prospective performance estimates, averaged across hemispaces and (ii) Posterior prospective performance estimates on the contralesional hemispace. Scores were inversed in order to have higher number corresponding to lower deficits. It is to note that these VLSM analyses were exploratory by nature, as with the relatively small sample size (*n* = 26) we have in the present study, we were unlikely to have enough power for corrected results and hence our criteria for multiple comparisons were not very strict (10% Overlap and using 1% False Discovery Rate correction for multiple comparisons). We report in the main results only the significant findings from these exploratory analyses (regions with *Z* > 2.363). Full lesion mapping methods (Supplementary methods 2), an overlay and VSLM analyses based on clinical AHP scores (Supplementary Results 4, Supplementary Fig. 4, Supplementary Table 1) are described in Supplementary materials.

### Data availability

The data that support the findings of this study are available on the Open Science Framework (https://osf.io/kwrnc/).

## Results

### Prior prospective performance estimates in AHP versus HP patients

**Figure 2:**
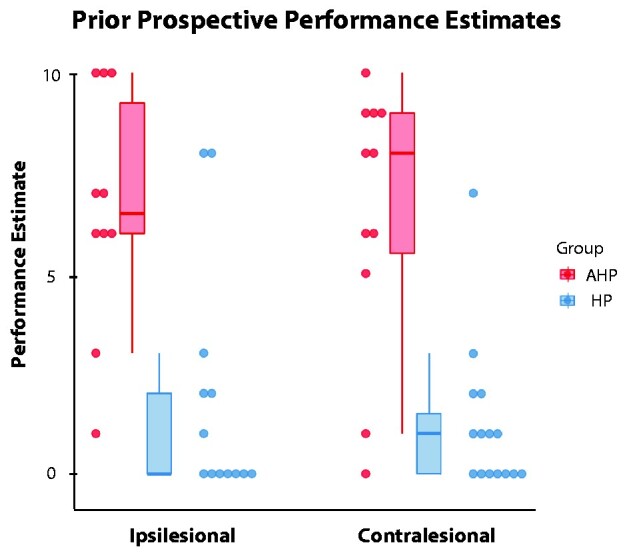
**Prior prospective performance estimates, in each hemispace (contralesional = left and ipsilesional = right).** Patients with Anosognosia for Hemiplegia (AHP in red) and Hemiplegia (HP in blue). Dots represent individual performance estimates. It is to note that due to the nature of the ‘Performance’ scale (10 = patients estimate they performed very well the task) and patients’ deficits (they could not perform the task, their estimates should be 0 = not performed at all), the higher the ratings, the more patients are being over-optimistic on their performance estimates, and being less aware of their deficit (i.e more anosognosic).

Before attempting to execute bimanual actions, AHP patients overestimated their ability to perform bimanual actions significantly more than HP patients (*Z* = −3.336, *P* < 0.001, ɳ_p_^2^ = 0.428, see Fig. 2). As expected, the hemispace where the items were presented had no effect on patients’ prior prospective estimates (*Z* = −0.144, *P* = 0.885, ɳ_p_^2^ = 0.001). Moreover, in testing the interaction between group and hemispace, we observed that group had no effect on the difference between ipsilesional and contralesional prior prospective estimates (*Z* = −1.369, *P* = 0.181, ɳ_p_^2^ = 0.072). Moreover, we found that the more unaware patients were on our clinical test of AHP (Feinberg awareness scale), the higher (unrealistic) baseline prospective performance estimate they gave on our task [r(23) = 0.590, *P* = 0.003; see Supplementary Fig. 3]. Taken together, these results point to the validity of our experimental set-up to capture anosognosic beliefs in AHP.

### Retrospective estimates in AHP patients

**Figure 3:**
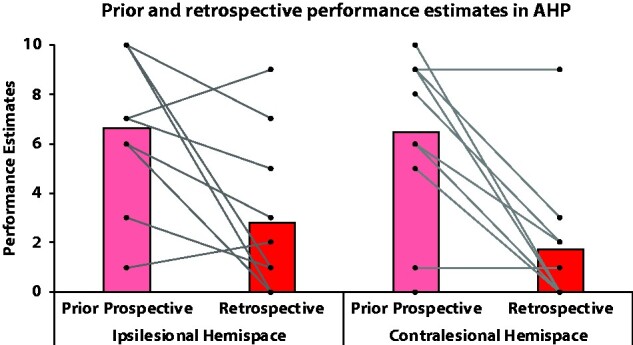
**Retrospective estimates. Prior and retrospective performance estimates in AHP patients, in each hemispace (ipsilesional = right hemispace vs. contralesional=left hemispace).** Performance estimates correspond to the estimates AHP patients gave on how well they think they performed the task, with 0 = not at all and 10 = extremely well. Individual data are represented by connected dots. Please note that each dot can represent several patients, if several patients gave the same score. From these estimates, we can observe that overall patients observed their failure to perform the action, after attempting to do the bimanual action (i.e. retrospective estimates being closer to the accurate score, 0 = failure to perform, than their prior prospective estimate). More precisely: (i) on the ipsilesional hemispace, only two patients scored higher retrospectively, however, one had a low prior estimate, suggesting baseline awareness at the time of testing; (ii) on the contralesional hemispace, only one patient scored higher retrospectively and two had low scores in both prior and retrospective.

After attempting to execute bimanual actions, most (although not all), AHP patients were able to recognize that they had failed to perform the action, and rated their ability to perform the action (retrospective performance estimates) as lower than prior to execution [prior performance estimate; *F*(1,9) = 19.916, *P* = 0.002, ɳ_p_^2^ = 0.689]. However, this recognition of failure did not significantly differ between hemispaces [*F*(1,9) = 0.330, *P* = 0.580, ɳ_p_^2^ = 0.035], and no interaction between time of the estimate and hemispaces was detected [*F*(1,9) = 0.378, *P* = 0.554, ɳ_p_^2^ = 0.040]. These results suggest that on average AHP patients were able to perceive their failure to perform bimanual tasks on both hemispaces, despite their initial anosognosic prospective estimates about their abilities on the same tasks and despite their neglect (Fig. 3; see Supplementary materials 3, for additional results).

### Posterior prospective estimates in AHP patients

**Figure 4:**
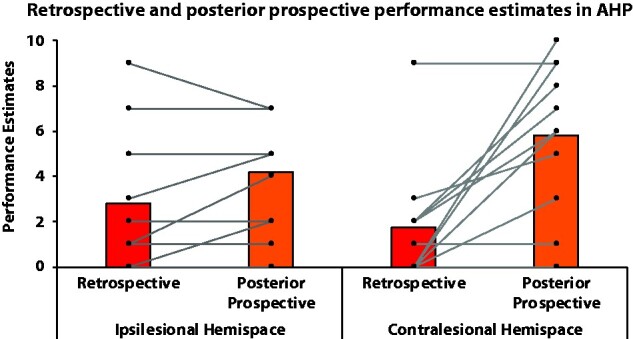
**Posterior prospective estimates: Retrospective and posterior prospective performance estimates in AHP patients, in each hemispace (ipsilesional = right vs. contralesional = left).** Performance estimates correspond to the estimates AHP patients gave on how well they think they performed the task, with 0 = not at all and 10 = extremely well. Individual data are represented by connected dots. Please note that each dot can represent several patients, if they gave the same score. From these estimates, we can observe: (i) on the ipsilesional hemispace, the majority of the patients stay close to their observation of failure to perform the task (retrospective estimate), with only 3 patients increasing their estimates (i.e. increasing their unawareness of their motor deficits); (ii) on the contralesional hemispace, the majority of the patients increase their performance estimates, going away from the observation of their failure to perform the task, and becoming less aware of their motor deficits. Only 3 patients retained information from their observation (same estimate for retrospective and posterior prospective estimates), with two patients recognizing and learning their motor deficit.

Overall, as expected given their high priors, AHP patients’ prospective posterior performance estimates were on average higher than their retrospective performance estimates [*F*(1,9) = 8.313, *P* = 0.018, ɳ_p_^2^ = 0.480], with no main effect of hemispace [*F*(1,9) = 0.197, *P* = 0.668, ɳ_p_^2^ = 0.021]. Crucially, there was an interaction in that how much patients’ posterior prospective performance estimates deviated from their retrospective performance estimate depending on which hemispace the action was performed in [*F*(1,9) = 5.711, *P* = 0.040, ɳ_p_^2^ = 0.388]. Indeed whereas there was no significant difference between their retrospective and posterior prospective estimates in the ipsilesional hemispace [*t*(9) = −1.463, *P* = 0.177], in the contralesional hemispace patients posterior prospective estimates were significantly higher from their retrospective estimates [*t*(10) = −3.870, *P* = 0.003], suggesting that most AHP patients failed to take into account their retrospective estimates, especially in the contralesional hemispace (Fig. 4).

### Bayesian, precision-weighted posterior beliefs in AHP

**Figure 5:**
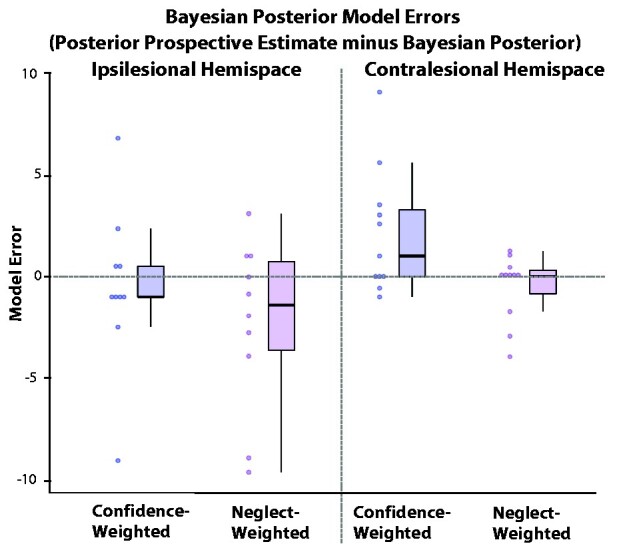
**Bayesian Posterior Model errors.** Computed as the difference between the Posterior Prospective Performance Estimate AHP patients gave and their Bayesian Posterior; computed either with their confidence ratings or their neglect scores as uncertainty, and thus in each hemispace (see section *Bayesian Posterior Beliefs* in the Methods section). Dots represent individual data.

When running one sample Wilcoxon Signed Rank Tests for each of our two Bayesian Posterior Model Errors (see Methods) in each hemispace (comparison to a median = 0, no significant error), we found that only the Confidence-Weighted Bayesian Posterior Model Error in the contralesional hemispace was significantly different from 0 [Ipsilesional Hemispace—Confidence-weighted Model Error: *t*(9) = −0.873, *P* = 0.383, BF_10_ = 0.335; Neglect-Weighted Model Error: *t*(9) = −1.376, *P* = 0.169, BF_10_ = 0.943; Contralesional Hemispace: – Confidence-weighted Model Error: *t*(10) = 2.033, *P* = 0.042, BF_10_ = 1.804; Neglect-Weighted Model Error: *t*(10) = −0.420, *P* = 0.674, BF_10_ = 0.461]. This suggests that—in terms of modelling Bayesian belief-updating—the only model that fails to explain belief-updating is the model using subjective confidence as a proxy for aberrant precision when assimilating evidence from the contralesional hemispace (Fig. 5).

Interestingly in the contralesional hemispace, comparing the model errors obtained with the confidence versus the neglect scores yielded a significant difference [*t*(10) = −3.274, *P* = 0.008, BF_10_ = 6.986], suggesting that the Neglect-Weighted Model is closer to the actual Prospective Posterior Performance estimate in the contralesional hemispace. Taken together, these results suggest that while both subjective confidence and visuospatial neglect affect belief-updating in AHP, in the contralesional hemispace this updating is best explained, in terms of Bayesian belief-updating, when using neglect as a proxy for precision.

### Lesion mapping results

#### Damaged areas related to deficits in prior prospective performance estimate

**Figure 6:**
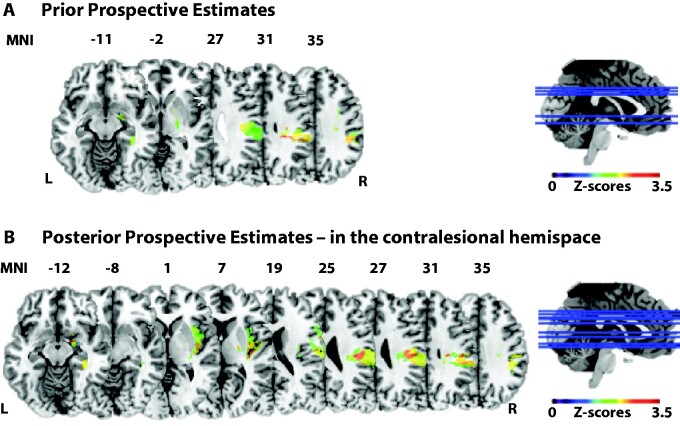
**Damaged areas related to Anosognosia for hemiplegia based on prospective performance estimates.** (**A**) Based on Prior Prospective Performance Estimates (inversed Prior Prospective Estimates averaged across hemispaces were entered as continuous predictor). (**B**) Based on Posterior Prospective Performance Estimates in the contralesional hemispace. Scores were inversed in order to have higher number corresponding to less deficit.

The VLSM analysis using the prior prospective performance estimate as predictor (inversed score) revealed a large cluster in the supra marginal gyrus area, but also lesions to the Pallidum, Hippocampus and Amygdala; and lesions of white matter tracts in portions of the SLF, Posterior and Superior Corona Radiata, as well as the posterior limb of the internal capsule (Fig. 6A, Supplementary Table 2A).

#### Damaged areas related to deficits in posterior prospective performance estimates

VLSM analysis using the Posterior Prospective Performance estimate in the contralesional hemispace as predictor identified large cluster lesions in Heschl’s gyri and the Insula but also the Postcentral sulcus and supra marginal gyrus, as well as clusters in the putamen, Amygdala, Hippocampus, Rolandic operculum and Superior Temporal areas; and lesions of white matter tracts in portions of the SLF, superior and posterior corona radiata (Fig. 6B; Supplementary Table 2B).

## Discussion

In the present study, we attempted to address outstanding questions regarding the inability of patients with AHP to update their beliefs about their motor abilities, despite the severe disabilities caused by stroke. We hypothesized that AHP patients would have greater difficulties in monitoring their errors retrospectively and updating their beliefs prospectively in the affected than the unaffected hemispace, where their inattention would render prediction errors imprecise and would thus influence the relative precision of prior beliefs and sensory prediction errors.

First at the behavioural level, we found that the retrospective performance ratings of most (but not all) patients with AHP suggested that they were able to recognize that they had failed to perform the attempted action, despite their more unrealistic prior beliefs about similar, motor abilities. This is a well-documented phenomenon in AHP research called emergent awareness^9^^,^^10^^,^^36^^,^^45^^,^^54^^,^^67^^,^^68^ and known to have some beneficial, therapeutic value.^69^ However, to our knowledge, differences in emergent awareness between the neglect-affected and non-affected hemispace has not been previously tested in patients with AHP. Contrary to our prediction, there were no hemispace differences in AHP patients’ retrospective performance estimates, suggesting that visuospatial neglect did not influence their sensorimotor monitoring at this level. In addition, retrospective-updating measures did not correlate with individual, neuropsychological neglect measures (see Supplementary results 3). It appears that the reduced attention spontaneously allocated to the contralesional hemispace by patients suffering from neglect did not prevent them from monitoring their errors when attention was experimentally drawn to that hemispace. It should however be noted that this is a partial finding as visuospatial neglect is a complex, multifactorial deficit with additional facets such as object-based frames of reference and different representational levels that were not assessed in the present study. Moreover, to keep this bedside experiment simple for our acute patients, our hemispace manipulations were based simply on controlling where the objects were presented and manipulated in space, rather than restricting patients’ vision, or tracking their eye movements. We have however taken individual, neuropsychological neglect measures in our patients and we have further used these in our modelling results, as discussed below.

Most interestingly, we observed that visual hemispace did have an effect in the degree to which the posterior prospective estimates of bimanual motor abilities of our AHP patients differed on average from their retrospective estimates of motor performance. Specifically, in the contralesional hemispace, patient’s posterior prospective estimates were significantly different from their retrospective estimates (showing more anosognosia), while there was no such difference in the ipsilesional hemispace. This finding suggests that in the contralesional hemispace, most AHP patients cannot ‘transfer’ their retrospective insight about observed motor failures to prospective beliefs about motor ability. Instead, these posterior beliefs seem closer to their unrealistic prior beliefs in the same hemispace. These results point towards a counterintuitive yet crucial finding, namely the visuospatial hemifield in which errors occur may affect prospective, belief-updating (Can I put on gloves?), without affecting retrospective, sensorimotor monitoring (How well did I put on gloves in this attempt?). In other terms, in the contralesional hemifield anosognosic patients can acknowledge their performance errors (complete failure due to the HP) to a degree, but they cannot use such observations to update their more general, prospective beliefs about their motor abilities. In clinical terms,^67^ these results suggest that in the contralesional hemispace, anticipatory awareness (i.e. prospective performance estimates) is not as influenced by emergent awareness (i.e. retrospective estimates) to the same degree as in the ipsilesional hemispace.

Importantly, we found that patients’ posterior performance estimates in the contralesional hemispace could be better explained by approximating precision with (objective) contralesional neglect, rather than by their ratings of subjective confidence. Under the assumption that our patients were ideal Bayesian observer^70^—but updating their beliefs with suboptimal precision—this aberrant precision is best reflected in objective measures of neglect. This was expected given that precision in this setting is a subpersonal estimate of uncertainty or reliability—as opposed to a declarative or subjective estimate. Subjective confidence in prior beliefs versus sensory information did however capture ipsilesional prospective beliefs, and patients with AHP appeared to have less confidence than HP patients in retrospective estimates and hence future studies should explore the contribution of subjective uncertainty to anosognosia.

Exploratory (given our sample size) lesion analyses revealed that anosognosic difficulties in belief-updating were associated with disruptions in tracts of the ventral attentional network (i.e. SLF connections between temporo-parietal junction and ventral frontal cortex, including in this case lesions to the insula, in line with previous studies).^50^ Lesions to the SLF were found for both prior and posterior prospective estimates, thus being involved in forming, prospective, counterfactual estimates about one’s motor abilities. Moreover, prior prospective estimates (i.e. learned counterfactual beliefs) were associated with lesions in the limbic regions (amygdala, hippocampus, pallidum), as previously hypothesized,^24^^,^^35^ while posterior prospective beliefs in the contralesional hemifield were also associated with lesions in the postcentral sulcus, inferior parietal cortex and superior temporal regions. Interestingly, we have also found associated lesions to these temporoparietal junction areas, as well as to the inferior and middle frontal gyri, with allocentric, mentalization deficits in AHP.^13^ This would suggest that anosognosic patients do not correct their unrealistic self-beliefs as they may be unable to take an allocentric stance on themselves, i.e. integrate their first-person experience of the body with third-person views to form a more ‘objectified’, counterfactual view of the self.^24^

In the current study, we find that similar lesions, as well as disconnections of these temporoparietal areas from their ventral frontal cortex connections via the SLF, lead also to failures to update counterfactual beliefs beyond the ‘here-and-now’ of sensorimotor experience. Indeed, different social or spatial perspectives were not at stake in the present experiment but patients were asked to use their motor, bimanual performance as it occurred in particular time and hemispace (‘Did you achieve this task here and now?’) to infer their corresponding motor abilities in a prospective manner which entails consideration of many possible (counterfactual) times and spaces (‘How well will you be able to achieve this task at home, or at work, tomorrow or next week?’). Thus, in this sense, our findings portray that the aforementioned lesions and disconnections affect patients’ ability to use sensory error information from the contralesional hemispace to draw more abstract, conclusions about self-related counterfactuals. While it is known that such ventral lesions may lead to a kind of ‘motivational’ neglect, or a difficulty to reorient attention in contralesional hemispace based on salience and behavioural relevance,^51^^,^^52^ the present association of hemispatial neglect and anosognosic beliefs (rather than just misperceptions) is novel. However, this finding is reminiscent of rare observations made by Mesulam (p. 1329)^51^ regarding the relationship between neglect and motivational expectations; ‘*Patients with unilateral neglect devalue the left side of the world and behave not only as if nothing is actually happening in the left but also as if nothing of any importance could be expected to emanate from that side*’. The current study indeed confirms that this observation applies also at the level of belief formation, so that even when patients are able to observe what has happened in the left hemispace (their motor errors), they do not experience such errors as ‘important enough’ beyond the given context to update their more abstract beliefs about their self. Or, as one of our patients said, ‘I know I can put on gloves by myself, I just could not do it now. If we were at home, this would be no problem’.

Indeed, we propose that the delusional aspects of anosognosia are best explained as the failure to evaluate the salience, or relevance of context-dependent sensorimotor errors (they occur in specific time and space) to more abstract (context-independent; they can refer to any time and space) beliefs about the self. Typically, errors occurring in the neglected hemispace and disconnections in the right salience network seem to result in patients being unable to assimilate the information from that space appropriately. Ultimately, they fail to integrate their sensorimotor errors from that space with other beliefs about their counterfactual self. This interpretation is also consistent with prior findings regarding the disruption and disintegration of several phenomenological and cognitive aspects of self-processing following damage to the temporo-parietal region, including self-reduplication and out-of-body experiences.^71^ The exact relationship between the counterfactual belief-updating impairment we examined in the present study and similar deficits in ‘allocentric’ mentalisation^13^ and weak central coherence^72^^,^^73^ that have been associated with similar multimodal integration networks, needs to be determined in future studies.

We wish to highlight, however, that we do not consider the disconnections and deficits measured in the present study to provide a full account for anosognosia, given the well-documented heterogeneity of the syndrome. Moreover, our data showed better belief-updating in the ipsilesional than in the contralesional hemispace but anosognosia and aberrant counterfactual beliefs were present in both hemifields, as it is long known clinically. Future studies should test further hemifield manipulations and could also investigate how different dimensions of neglect influence unawareness of deficit. Furthermore, on top of other limitations mentioned above, we wish to highlight intrinsic limitations of the present study: the limited number of patients as anosognosia for HP is a relatively rare phenomenon, the number of trials per condition, and the effect of lesion’s hemisphere, as we focussed on right hemisphere stroke patients. Future studies should replicate and extend the present findings in a bigger sample, taking into account more interindividual variability, such as neglect, as well as laterality effects.

In brief, our study suggests that precision-based, belief-updating deficits may also contribute to the aetiology of the AHP syndrome, and particularly its delusional features that have received less experimental attention than its sensorimotor features in the past. Our study also has wider implications for understanding ‘counterfactual’ belief-updating, self-awareness and prospective metacognition in health, as well as in many other pathologies with awareness or insight deficits. Finally, future rehabilitation studies should explore whether feedback about one’s paralysis is best offered on the ipsilesional hemispace.

## Supplementary material

Supplementary material is available at *Brain Communications* online.

## Supplementary Material

fcab098_Supplementary_Data

## Abbreviations

AHP =: anosognosia for hemiplegia
HP =: hemiplegia
SLF =: superior longitudinal fasciculus
SMG =: supra marginal gyrus
VLSM =: voxel-based lesion symptom mapping
