# Supplementary material for: Updating beliefs beyond the here-and-now: the counter-factual self in anosognosia for hemiplegia
Source: Brain Commun. 2021 May 21;3(2):fcab098. doi: 10.1093/braincomms/fcab098 (PMC8209286; doi:10.1093/braincomms/fcab098)
Supplement: fcab098_Supplementary_Data [file fcab098_supplementary_data.pdf]

SUPPLEMENTARY MATERIALS

Supplementary Methods

1. AHP vs. HP classification

Patients were divided into two groups based on the presence (AHP group, N = 11) or absence (HP group, N = 15) of AHP, diagnosed as in previous studies[1,2] based on the Berti interview[3] and validated using the Feinberg scale.

This classification was based on the Berti, Ladavas, and Della Corte interview,[3] which includes general questions (e.g., 'why are you in the hospital?'), followed by specific questions regarding motor ability (e.g., 'Can you move your left arm?'), and 'confrontation' questions (e.g., 'Please touch my hand with your left hand. Have you done it?'). The interview is scored on a 3-point scale (2=denial of motor impairment and failure to reach the examiners hand; 1=denial of motor impairment but admits to failure to reach examiner hand; and 0=full acknowledgement of motor deficits), with patients scoring 1 or 2 categorised as anosognosic. The Feinberg, Roane, and Ali scale[4] was used as a secondary measure of unawareness. The scale consists of 10 items including general self-report items (e.g. 'Do you have any weakness anywhere?') and task-related items (e.g., 'Please try and move your left arm for me. Did you move it?'). Responses were scored by the examiner for each item (0=completely aware, .5=partially unaware, and 1=complete unawareness), and summed to produce an overall 'Feinberg awareness score' (0=complete awareness, 10=complete unawareness).

2. Lesion mapping

Routinely acquired clinical scans obtained on admission (<2 days post stroke) were collected (21 via computerised tomography, CT; and 5 via magnetic resonance imaging, MRI). Lesions from these scans were segmented and co-registered using the following, expert manual procedure, which remains the best method to date for lesion mapping of CT scans and shown to be more accurate than automatized methods.[5-7] Lesions were outlined by two expert clinicians (V.M. and S.B) who were blind to each scan's group classification. In the case of disagreement of two lesion plots, the opinion of a third, expert anatomist was requested. Scans were registered to the T1-weighted MRI scan template (ICBM152) of the Montreal Neurological Institute,

furnished with the MRIcron software (ch2, http://www. cabiatl.com/mricro/mricron/index.html). First, the standard template (size: 181 x 217 x 181 mm, voxel resolution: 1 mm2) was rotated to match the orientation of the patient's MRI or CT scan. Lesions were outlined on the axial slices of the rotated template. The resulting lesion volumes were then rotated back into the canonical orientation, to align the lesion volumes of each patient to the same stereotaxic space. Finally, to exclude voxels of lesions outside white and grey matter brain tissue, lesion volumes were filtered by means of custom masks based on the ICBM152 template.

Lesion volume analysis was computed with Non-Parametric Mapping (NPM) software,[8] in order to ensure no difference between groups that could explain lesion and behavioural results.

First, a lesion overlap was calculated to create a color-coded overlay map of injured voxels across patients in each group to provide an overview of all lesioned brain areas in each group (AHP and HP, see Supplementary Fig.4). In order to investigate the neural correlates of AHP, we first compared the lesion maps of the two groups by means of a subtraction technique and a voxel-based lesion comparison based on the Libermeister binomial test (False discovery rate FDR corrected)[9]. The outcome of the subtraction and the significant lesion maps of the voxel-based lesion techniques were superimposed onto T1 templates (AAL) to calculate the number of lesioned voxels in cerebral areas and the centre of the mass in each damaged area. Statistical contribution of lesion location to deficit in general motor awareness was also tested using voxel-based lesion symptom mapping[10] (VLSM), with inversed Feinberg scores[4] entered as continuous predictor (Supplementary Fig.4, Supplementary Table 1).

All lesion results were visualised in MRIcron. Three anatomical templates served to identify grey and white matter region labels: the “automated anatomical labelling” (AAL) template,[11] the JHU white-matter tractography atlas,[12] and the “NatBrainLab” template of the “tractography based Atlas of human brain connections Projection Network”.[13,14]

Supplementary Results

1. Importance Ratings

To ensure basic perception and relevance of the experimental materials, the objects corresponding to bimanual everyday actions (e.g. using cutlery) were positioned on a table in front of the participants, on their midline, and they were asked to rate the importance of these actions in their everyday life (e.g. "How important is it for you to be able to cut your steak using both hands?"), on a scale from 0=not at all to 5=extremely important.

AHP and HP patients did not differ in how they rated the importance of being able to perform the actions (Z=-.286, p=.778, *d*=-0.120; AHP group: *M*=4.1, *SD*=0.738; HP group: *M*=4.192; *SD*=0.805). Overall patients found important to be able to perform these bimanual actions in their everyday life, independently of anosognosia.

2. Confidence Estimates

Confidence estimates were analysed separately at each time point. AHP patients were significantly less confident than HP patient only on the retrospective confidence estimate (prior prospective: Z=-1.717, p=.088, η^2=.113; retrospective: Z=-2.102, p=.035, η^2=.170; posterior prospective: Z=-1.482, p=.145, η^2=.084; see Supplementary Fig.1). Patients estimated their confidence levels similarly on both hemispaces**,** in any of the time points, suggesting that external spatial precision did not influence their internal precision as measured by confidence (prior prospective: Z=-1.422, p=0.176, η^2=.078; retrospective: Z=-.144, p=.922, η^2=.001; and posterior prospective confidence estimates: Z=-.715, p=.799, η^2=.02). Moreover, anosognosia level had no significant effect on the difference between the confidence estimates on the ipsilesional and the contralesional hemispace in any of the time points (prior prospective: Z=-.833, p=.424, η^2=.027; retrospective: Z=-.879, p=.397, η^2=.03; and posterior prospective confidence estimates: Z=-1.389 p=.170, η^2=0.074).

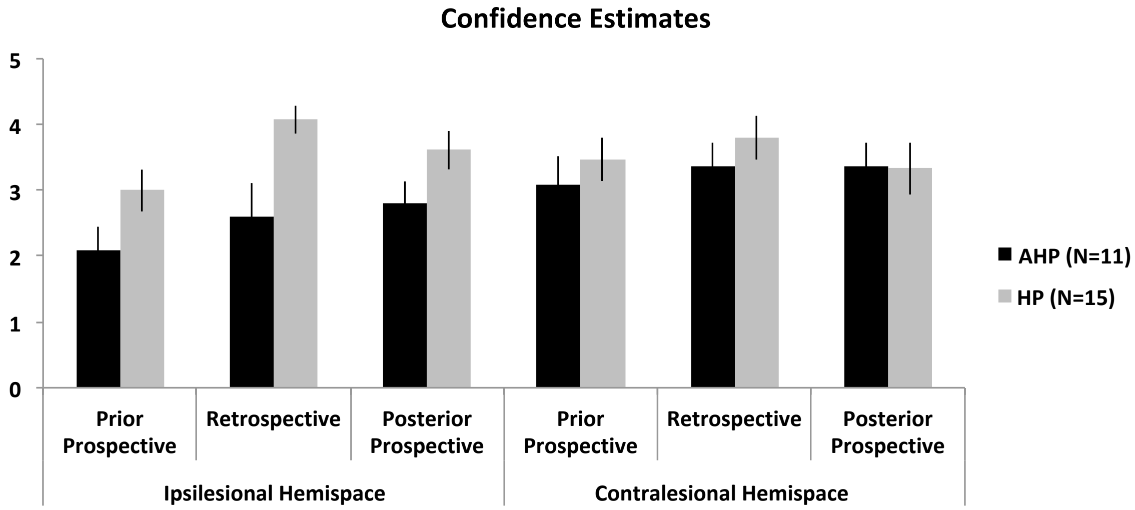

Supplementary Fig.1. Confidence Estimates, *at each time point, in each hemispace for patients with Anosognosia for Hemiplegia (AHP in black) and Hemiplegia (HP in grey). Error bars represent the standard error of the mean.*

3. Performance Estimates across AHP and HP patients

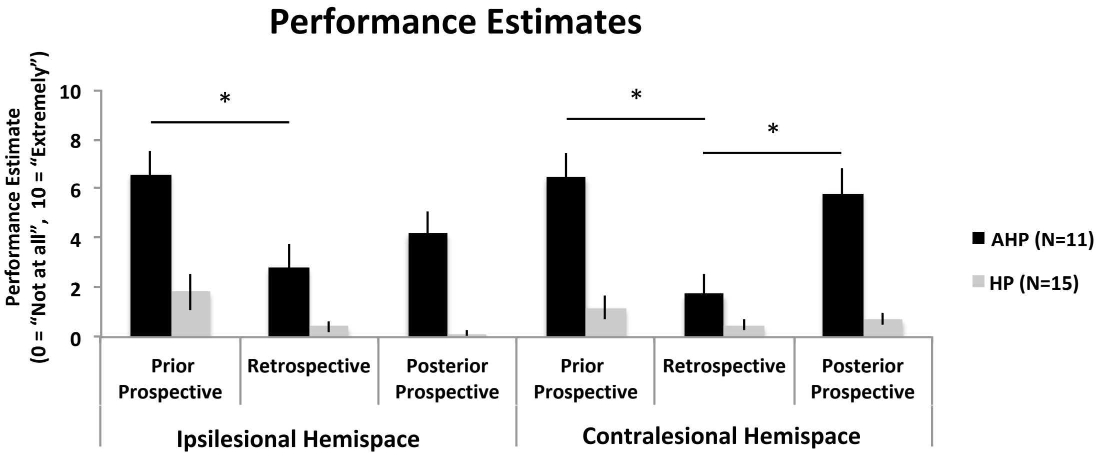

Supplementary Fig.2. Performance Estimates, *at each time point, in each hemispace (contralesional=left and ipsilesional=right). Patients with Anosognosia for Hemiplegia (AHP in black) and Hemiplegia (HP in grey). *denotes significant difference (p<0.05).*

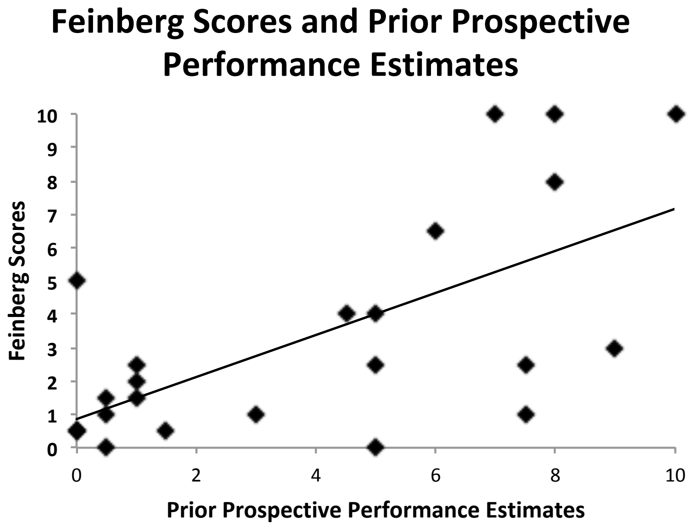

Supplementary Fig.3. *Correlation between Prior Prospective Performance Estimates and Feinberg assessment scores, including all patients (AHP and HP).*

Retrospective Updating in AHP and HP patients

To examine the degree to which patients were able to observe that they have failed to perform an action despite their unrealistic prior prospective estimates, we used a 'Retrospective Updating' index i.e. the difference between retrospective and prior prospective performance estimates for each patient.

Directly after an execution attempt, AHP patients updated their performance estimate more than HP patients (Z=2.404, p=.015, η^2=.222; Fig.3). This can in part be explained by the fact that AHP patients have a higher Prior Prospective performance estimate than HP, and HP patients' Prior and Retrospective estimates are close to 0. Patients updated their performance estimate after execution similarly on the ipsilesional and contralesional hemispace (Z=-.521, p=.623, η^2=.01). Group had a trend effect on the difference between ipsilesional and contralesional Retrospective Updating scores (Z=-1.958, p=.051, η^2=.147). We elected to follow-up this two-tailed probability, given our predictions of worse performance in the AHP group and the contralesional hemispace. However, when comparing across hemispaces in each group, we observed no significant difference in Retrospective Updating between the two hemispaces in either group (AHP: Z=-1.191, p=0.262, η^2=.055; HP: Z=-.775, p=.469, η^2=.023). These results suggest that contrary to our prediction, AHP patients were able to perceive their failure to perform bimanual tasks on both hemispaces, despite their initial anosognosic prospective estimates about their abilities on the same

tasks. Control patients were more realistic at the onset and were able to monitor their errors on both hemispaces.

Prospective Updating in AHP and HP patients

To generate a simple measure of how patients evaluate their bimanual abilities after noticing they have failed in a corresponding bimanual task (i.e. without taking confidence ratings into account), we calculated the difference between the posterior prospective and the retrospective performance estimates for each patient, as a posterior prospective update measure. AHP patients updated their performance estimate less than HP patients ($Z=-2.478$, $p=0.011$, $\eta^2=0.236$), i.e. AHP patients changed their opinion about their future abilities less than HP patients even after reporting that they failed to perform an action (see Supplementary Fig.2). Patients updated their prospective performance estimate after execution better on the ipsilesional than on the contralesional hemispace ($Z=-2.731$, $p=.005$, $\eta^2=0.287$). Group had a trend towards a significant effect on the difference between ipsilesional and contralesional posterior prospective update scores ($Z=-2.079$, $p=.072$, $\eta^2=0.166$). As above, we elected to follow up this 2-tailed probability given our predictions about worse performance in the target than in the control group, and in the contralesional hemispace. Bonferroni-corrected (alpha=0.025) revealed only a trend towards significant difference in the AHP group ($Z=-2,207$, $p=.031$, $\eta^2=0.187$) and no significant difference in the HP group (HP: $Z=-1.511$, $p=.250$, $\eta^2=0.088$).

To investigate whether the results found for the Retrospective and Prospective Updating indexes could be linked to neuropsychological performance, Spearman non-parametric correlations with anosognosia and neuropsychological scores were conducted. Both Retrospective and Prospective Updating on the contralesional hemispace negatively correlated with the awareness scores (Berti motor awareness score: $r(26)=-.543$, $p=.004$; $r(26)=-.519$, $p=.007$, Feinberg assessment scores: $r(26)=-.482$, $p=.020$; $r(26)=-.467$, $p=.025$; respectively), but with none of the neglect scores, nor other neuropsychological scores.

4. Lesion Mapping Supplementary Results

First, an analysis of the volume of the lesions was computed with Non-Parametric Mapping (NPM) software (Rorden et al., 2007) and showed no significant difference between AHP and HP patients ($Z=-1.272$, $p=.217$, $\eta^2=0.062$).

Damaged areas related to anosognosia for hemiplegia

In order to investigate the neural correlates of AHP, we first compared the lesion maps of the AHP and HP groups (Supplementary Fig.4A&B) by a voxel based lesion comparison based on the Libermeister binomial test (False discovery rate, FDR corrected). Using the more liberal cut-off for significant clusters (Z>2.363), numbers of regions appeared to be significantly more impaired in AHP than HP; clusters included temporal and postcentral areas as well as the Supramarginal Gyrus and Heschl (See Supplementary Fig.4D, and Supplementary Table 1A).

As an illustration, we also computed the subtraction map, showing regions more damaged in AHP patients than in HP patients (>30%; See Supplementary Fig.4C).

VLSM analysis using the inversed continuous Feinberg awareness scores, revealed that voxels within several regions including the amygdala, hippocampus, as well as the insula, the supramarginal, the angular and superior and mid-temporal gyrus (SMG, AG and STG), were associated with differences in awareness (see Supplementary Fig.4E and Supplementary Table 1.B).

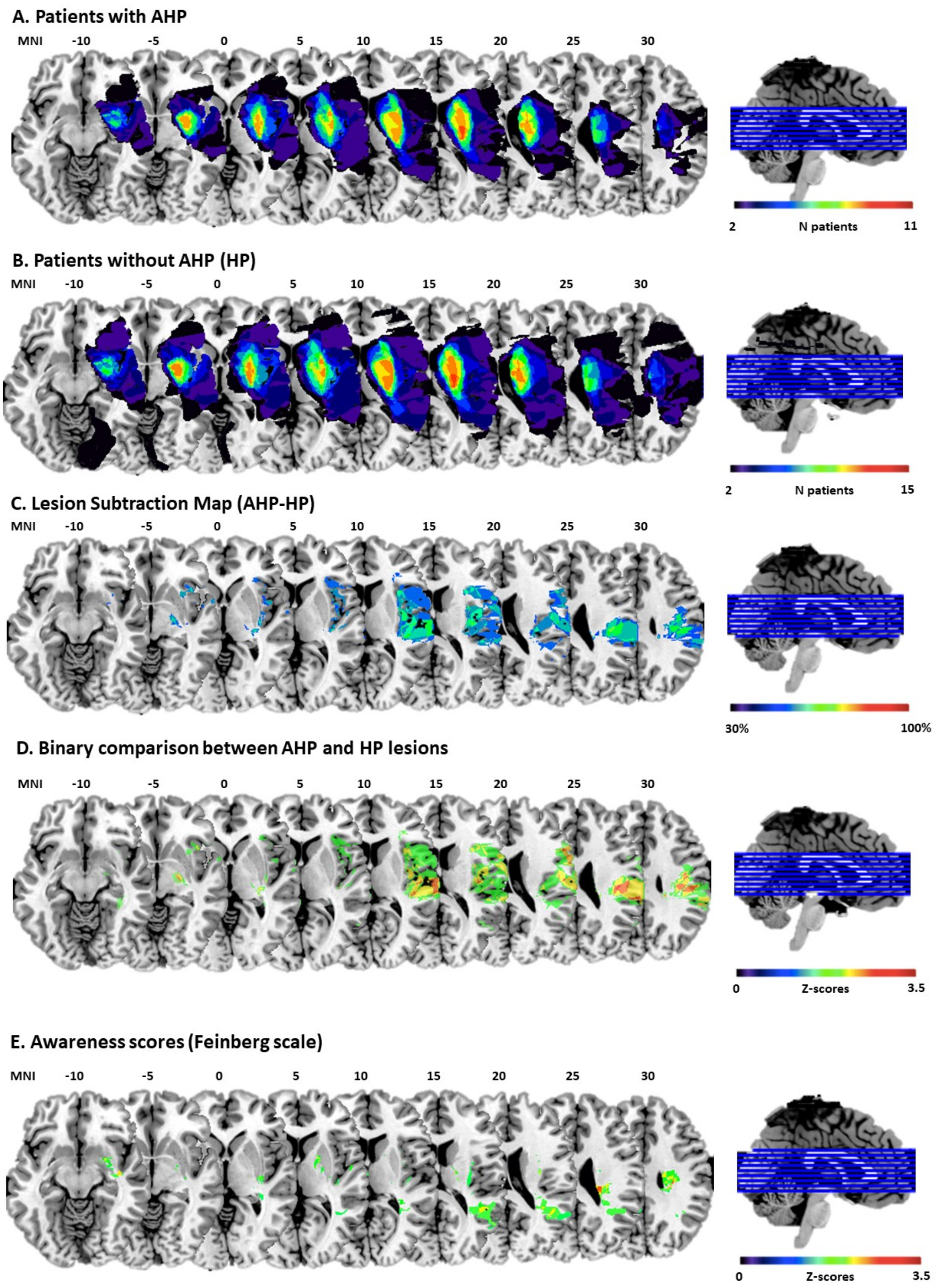

Supplementary Fig.4. Damaged areas related to Anosognosia for Hemiplegia. *A. Overlay of lesions in AHP patients (N=11). B. Overlay of lesions in HP patients (N=15). C. Lesion Subtraction Map (AHP-HP patients). D. Binary Comparison between AHP and HP lesions.*

E. Damaged MNI voxels predicting the severity of unawareness of symptom (Feinberg scale, inverted, continuous measure; p<.05 for Z>1.6449).

Supplementary Table 1. Damaged areas related to Anosognosia for Hemiplegia. A. Binary Comparison between AHP and HP lesions. B. Damaged MNI voxels predicting the severity of unawareness of symptom (Feinberg scale, inverted, continuous measure; p<.05 for Z>1.6449).

**A. Binary VLSM comparison between AHP and HP**						
**Region (AAL template)**	**N voxels**	**%N>0**	**Z peak**	**X**	**Y**	**Z**
Unclassified	4888	0.001	3.041	40	-23	25
Temporal_Sup	4996	0.198	3.208	69	-26	10
Postcentral	2165	0.071	3.041	46	-23	32
SupraMarginal	4018	0.255	3.041	47	-20	31
Heschl	1061	0.548	3.016	38	-22	12
Precentral	780	0.029	2.813	57	10	21
Frontal_Inf_Oper	3205	0.287	2.813	55	16	20
Insula	2992	0.212	2.730	34	-18	13
Rolandic_Oper	6125	0.571	2.633	61	0	14
Frontal_Inf_Tri	645	0.038	2.590	37	21	13
Hippocampus	359	0.047	2.406	17	-4	-15
Amygdala	218	0.111	2.406	22	-2	-12
Putamen	9	0.001	2.406	30	0	-8
Temporal_Pole_Sup	106	0.01	2.406	66	4	-2
Temporal_Mid	339	0.01	2.406	40	-55	18

**B. Awareness score (Feinberg scale)**						
**Region (AAL template)**	**N voxels**	**%N>0**	**Z peak**	**X**	**Y**	**Z**
Unclassified	4336	0.001	3.138	31	-5	-15
Amygdala	732	0.373	3.138	29	-5	-15
Hippocampus	934	0.123	2.759	33	-13	-14
Thalamus	15	0.002	2.432	18	-11	16
Temporal_Mid	1415	0.040	2.425	40	-53	18
Insula	336	0.024	2.387	33	-9	18
Pallidum	148	0.068	2.359	25	-6	-3
Temporal_Sup	550	0.022	2.265	48	-44	14
Heschl	178	0.092	2.177	50	-9	6
Putamen	53	0.006	2.138	33	-16	0
ParaHippocampal	70	0.008	1.947	22	4	-20
SupraMarginal	24	0.002	1.947	48	-44	24
Angular	204	0.015	1.947	46	-48	22
Temporal_Pole_Sup	26	0.002	1.947	28	8	-19
Rolandic_Oper	3	0.000	1.791	54	-2	6

Supplementary Table 2. Number of significant voxels (from the atlas of grey matter – AAL – and white matter – JHU – and NatBrainLab's atlas) resulting from the VLSM analyses. A. Using Prior Prospective Performance Estimates as predictor (averaged across hemispaces). B. Using Posterior Prospective Performance Estimates in the contralesional as predictor. Only voxels with Z>2.365 were considered; with 10% Overlap and using 1% False Discovery Rate correction for multiple comparisons.

**A. Prior Prospective Performance Estimates**							
	**Region**	**N voxels**	**%N>0**	**Z peak**	**X**	**Y**	**Z**
AAL	Unclassified	3678	0,001	3,305	32	-24	29
	SupraMarginal	3277	0,208	2,544	47	-37	31
	Pallidum	176	0,080	2,508	20	2	-2
	Hippocampus	67	0,009	2,429	22	-7	-11
	Amygdala	38	0,019	2,429	23	-7	-11
JHU	Unclassified	7002	0.001	3.305	28	-29	30
	Posterior_coronaadiata	134	0.036	3.305	27	-29	30
	Superior_longitudinal_fasciculus	886	0.134	3.305	32	-24	29
	Superior_coronaadiata	74	0.010	2.457	27	-21	33
	Posterior_limb_of_internal_capsule	123	0.033	2.429	12	-5	8
Natbrain	Unclassified	5931	0.002	3.305	31	-27	30
	Arcuate_Anterior_Segment	776	0.093	3.305	32	-24	29
	Cortico_Ponto_Cerebellum	60	0.012	3.305	27	-27	30
	Cortico_Spinal	872	0.040	3.305	27	-29	30
	Internal_Capsule	240	0.014	3.036	25	-30	30
	Corpus_Callosum	22	0.000	2.963	24	-30	30
	Inferior_Longitudinal_Fasciculus	238	0.017	2.481	26	-14	-6
	Opticadiations	94	0.026	2.481	25	-13	-6
	Anterior_Commissure	103	0.019	2.429	22	-7	-11

**B. Posterior Prospective Performance estimate in the contralesional hemispace**							
	**Region**	**N voxels**	**%N>0**	**Z peak**	**X**	**Y**	**Z**
AAL	Unclassified	5326	0.001	3.19	23	-13	-4
	Heschl	821	0.424	3.89	38	-22	7
	Insula	3668	0.259	3.38	45	10	1
	Putamen	95	0.011	3.37	30	0	-8
	Postcentral	3022	0.098	3.09	46	-23	32
	SupraMarginal	3778	0.239	3.09	47	-20	31
	Amygdala	165	0.083	2.88	22	-2	-12
	Hippocampus	249	0.032	2.84	18	-6	-12
	Rolandic_Oper	2159	0.201	2.67	54	-16	19
	Caudate	3	0.0003	2.58	18	-16	25
	Temporal_Sup	376	0.014	2.52	41	-14	1
JHU	Unclassified	18664	0.003	3.891	38	-22	7
	External_capsule	160	0.029	3.371	30	0	-8

	Superior_longitudinal_fasciculus	1200	0.182	3.098	39	-22	25
	Superior_corona_radiata	203	0.027	3.023	28	-22	30
	Posterior_corona_radiata	210	0.057	2.903	26	-24	29
	Posterior_limb_of_internal_capsule	59	0.016	2.848	19	-16	-4
	Cerebral_peduncle	14	0.006	2.848	19	-16	-5
	Retrolenticular_part_of_internal_capsule	111	0.045	2.485	36	-24	-3
Natbrain	Unclassified	16396	0.005	3.891	38	-22	7
	Inferior_Occipito_Frontal_Fasciculus	205	0.016	3.371	30	0	-8
	Cortico_Spinal	795	0.036	3.195	23	-13	-4
	Opticadiations	332	0.091	3.105	24	-14	-5
	Arcuate_Anterior_Segment	2103	0.251	3.098	40	-23	25
	Internal_Capsule	169	0.010	3.055	20	-13	-4
	Anterior_Commissure	245	0.046	2.882	22	-2	-12
	Inferior_Longitudinal_Fasciculus	341	0.024	2.848	23	-9	-11

Supplementary Materials References

1. Moro V, Pernigo S, Tsakiris M, Avesani R, Edelstyn NM, Jenkinson PM, Fotopoulou A. Motor versus body awareness: voxel-based lesion analysis in anosognosia for hemiplegia and somatoparaphrenia following right hemisphere stroke. *Cortex* 2016; 83:62-77.

2. Besharati S, Forkel SJ, Kopelman M, Solms M, Jenkinson PM, Fotopoulou A. Mentalizing the body: spatial and social cognition in anosognosia for hemiplegia. *Brain*. 2016; 139(3):971-85.

3. Berti A, Làdavas E, Della Corte M. Anosognosia for hemiplegia, neglect dyslexia, and drawing neglect: Clinical findings and theoretical considerations. *Journal of the International Neuropsychological Society* 1996; 2(5): 426-40.

4. Feinberg TE, Roane DM, Ali J. Illusory limb movements in anosognosia for hemiplegia. Journal of Neurology, *Neurosurgery & Psychiatry.* 2000; 68(4):511-3.

5. Maier O, Schröder C, Forkert ND, Martinetz T, Handels H. Classifiers for ischemic stroke lesion segmentation: a comparison study. *PloS one.* 2015; 10(12):e0145118.

6. Liew SL et al. A large, open source dataset of stroke anatomical brain images and manual lesion. *Earth System Science Data*. 2018; 5:180011.

7. de Haan B, Karnath HO. A hitchhiker's guide to lesion-behaviour mapping. Neuropsychologia. 2018;115:5-16.

8. Rorden C, Karnath HO, Bonilha L. Improving lesion-symptom mapping. *Journal of cognitive neuroscience.* 2007; 19(7):1081-8.

9. Benjamini Y, Hochberg Y. Controlling the false discovery rate: a practical and powerful approach to multiple testing. *Journal of the Royal statistical society: series B (Methodological)*. 1995; 57(1):289-300.

10. Bates E, Wilson SM, Saygin AP, Dick F, Sereno MI, Knight RT, Dronkers NF. Voxel-based lesion–symptom mapping. *Nature neuroscience*. 2003; 6(5):448-50.

11. Tzourio-Mazoyer N, Landeau B, Papathanassiou D, Crivello F, Etard O, Delcroix N, Mazoyer B, Joliot M. Automated anatomical labeling of activations in SPM using a macroscopic anatomical parcellation of the MNI MRI single-subject brain. *Neuroimage*. 2002;15(1):273-89.

12. Mori S, Wakana S, Van Zijl PC, Nagae-Poetscher LM. MRI atlas of human white matter. Elsevier; 2005.

13. Catani M, de Schotten MT. Atlas of human brain connections. Oxford University Press; 2012.

14. de Schotten MT, Bizzi A, Dell'Acqua F, Allin M, Walshe M, Murray R, Williams SC, Murphy DG, Catani M. Atlasing location, asymmetry and inter-subject variability of white matter tracts in the human brain with MR diffusion tractography. *Neuroimage*. 2011;54(1):49-59.
